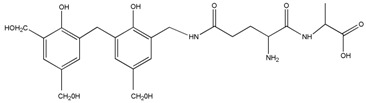# Study on the Soy Protein-Based Wood Adhesive Modified by Hydroxymethyl Phenol

**DOI:** 10.3390/polym8070256

**Published:** 2016-07-12

**Authors:** Hong Lei, Zhigang Wu, Ming Cao, Guanben Du

**Affiliations:** 1 Yunnan Provincial Key Laboratory of Wood Adhesives and Glued Products, Southwest Forestry University, Kunming 650224, Yunnan, China; wzghappy@swfu.edu.cn (Z.W.); caominghappy@swfu.edu.cn (M.C.); 2 Material Science and Technology College, Beijing Forestry University, Beijing 100083, China

**Keywords:** hydroxymethyl phenol, soy-based adhesive, cross-linker, dipeptide

## Abstract

To explain the reason why using phenol-formaldehyde (PF) resin improves the water resistance of soy-based adhesive, the performance of soy-based adhesive cross-linked with hydroxymethyl phenol (HPF) and the reaction between HPF and a common dipeptide *N*-(2)-l-alanyl-l-glutamine (AG) being used as a model compound were studied in this paper. The DSC and DMA results indicated the reaction between HPF and soy-based adhesive. The soy-based adhesive cross-linked with HPF cured at a lower temperature than the adhesive without HPF. The former showed better mechanical performance and heat resistance than the latter. The ESI-MS, FT-IR and 13C-NMR results proved the reaction between HPF and AG. Because of the existence of branched ether groups in the ^13^C-NMR results of HPF/AG, the reaction between HPF and AG might mainly happened between hydroxymethyl groups and amino groups under a basic condition.

## 1. Introduction

Today, the development of environmentally friendly wood adhesives is an important goal in the wood industry. Soy protein–based adhesive mainly prepared with soy flour is one of the successful bio-based environmentally friendly adhesives. It is reported that one soy-based adhesive has been used for the production of interior plywood panels since 2004 [1].

The main problem with soy-based adhesive is its poor water resistance. Much concern has been put on this issue. Considering soy proteins are polymerized with the amino acid units, the possible reactive groups in the soy protein structure mainly include –OH, –SH, –COOH and –NH_2_ groups. On the basis of the possible reactions with these reactive groups, some cross-linkers, such as melamine-formaldehyde resin [2,3], polyacrylic ester [4], epoxy [5], aldehyde and its derivatives [6,7], and so on, have been used to improve the water resistance of soy-based adhesives. As a widely-used wood adhesive for exterior wood panels, phenol-formaldehyde (PF) resin is reported by several research teams to be used as a cross-linker of soy-based adhesive [8,9,10]. The reason for the application of PF resin to improve the water resistance of the soy-based adhesive is generally thought to be the reaction between the PF and soy protein, especially the reaction between the hydroxymethyl groups of PF resin and the amino groups of soy protein. However, because of the complex structure of the kinds of amino acid units of soy protein, the study of the mechanism of the modification of soy-based adhesive with PF resin is very difficult. Additionally, once the soy-based adhesive is prepared with soy flour, a complicated mixture with multiple ingredients, it will be more difficult to explain the reason why PF resin should be used to improve the water resistance of soy-based adhesive.

To explore the reaction between the soy protein and PF resin, *N*-(2)-l-alanyl-l-glutamine (AG) with possible reactive groups –NH_2_ and –COOH, a common and comparatively cheap dipeptide, was chosen in this work as a model compound of soy protein to react with the PF resin. To guarantee the possible reaction between the soy protein and PF resin to a great degree, hydroxymethyl phenol (HPF), with more hydroxymethyl reactive groups prepared in our laboratory than a PF resin prepared under normal procedures, was used as a modifier of the soy-based adhesive for the analysis. The objectives of this work are then to shed light on the reaction between PF resin and soy protein and to be provide guidance for the application of soy-based adhesive cross-linked with PF resin. The study on the reaction between the PF resin and amino acids or peptides will not only be a breakthrough on the modification mechanism between PF resin and soy-based adhesive, but it will also be helpful for the analysis of the reaction between aldehyde and soy-based adhesive. On the basis of the understanding of the reaction between PF and soy-based adhesive, it is possible to find other non-toxic cross-linkers to replace PF resin to modify the soy-based adhesive. 

## 2. Materials and Methods

### 2.1. Materials

The defatted soy flour (53.4% protein content) was obtained from Yuxin Soybean Protein Co., Ltd., Binjiang, China. Formaldehyde 50 wt % were donated by Kunming Xinfeilin Panel Board Co., Ltd., Kunming, China. *N*-(2)-l-alanyl-l-glutamine (AG) was purchased from Chinese Medicine Group Chemical Reagent Co., Ltd., Shanghai, China, with a purity of 99%. 

### 2.2. Preparation of Hydroxymethyl Phenol

A 500 mL flat bottom flask equipped with a condenser, thermometer and a magnetic stirrer bar was charged with phenol and formaldehyde 50%. The molar ratio of phenol to formaldehyde was set at F/P = 3.0/1. 30% sodium hydroxide solution was added to adjust the pH to 8.5–9.0. The mixture was kept at 30 ± 1 °C for 24 h. Then, the HPF sample was gotten.

### 2.3. Preparation of Soy-Based Adhesive

Soy-based adhesive was prepared according to a method already reported [11]: In a three-neck round-bottom flask equipped with a mechanical stirrer, thermometer and condenser was charged with 320 parts water. Then 80 parts soy flour was charged to the rapidly stirring solution. The mixture was heated to 45 °C and 21.3 parts 30% sodium hydroxide solution was added. After stirring for 30 min, 20 parts 40% urea solution was added and was stirred for 20 min. The mixture was cooled to room temperature in an ice bath. The solid content of the resulted soy-based adhesive with a name of S was 23% ± 1%.

### 2.4. Preparation of HPF/AG

A 500 mL flat bottom flask equipped with a condenser, thermometer and a magnetic stirrer bar was charged with 40 g HPF and 1.0 g AG. Then 30% sodium hydroxide solution was added to adjust the pH to 8.0–9.0. The mixture was heated to 80 °C and kept at this temperature for 30 min. Then, the HPF/AG sample was gotten. The sample with the name of HPF’ was also prepared as control. It was prepared by heating at 80 °C for 30 min. 

To make sure the possible reaction between HPF and AG to be happened, the ratio between HPF to AG for ^13^C-NMR analysis is 40 g/16 g.

### 2.5. Differential Scanning Calorimetry (DSC)

The soy-based adhesive, HPF and their mixture was tested by a DSC 204 F1 spectrometer (NETZSCH Scientific Instruments Trading Ltd., Waldkraiburg, Germany). The mixture was composed of 100% soy -based adhesive and 12% HPF on a dry basis. All the DSC experiments were conducted under a heat rate of 10 °C/min. The testing temperature range was 30–250 °C. The software used for data treatment was PYRISTM Version 4.0.

### 2.6. Dynamic Mechanical Analysis (DMA)

The soy-based adhesive and its mixture with HPF were tested by DMA on a NETZSCH DMA-242 apparatus (NETZSCH Scientific Instruments Trading Ltd., Waldkraiburg, Germany). Triplicate samples of two poplar wood plies (each 1.5 mm thick) bonded with the mixture to form a sample dimensions of 50 mm × 10 mm × 3 mm were tested in a non-isothermal mode between 40 and 300 °C under a heat rate of 5 °C/min in a three-point bending mode on a span of 40 mm. The stress was 1.5 N and the frequency was fixed at 50 Hz. The software used for data treatment was NETZSCH Proteus.

### 2.7. Electrospray Ionization Mass Spectrometry (ESI-MS)

The spectra were recorded on a Waters Xevo TQ-S instrument (Waters, Milford, MA, USA). *N*-(2)-l-alanyl-l-glutamine, HPF, and their mixture samples were dissolved in chloroform respectively at a concentration of about 10 μL/mL and injected into the ESI source plus ion trap mass spectrometer via a syringe at a flow rate of 5 μg/s. Spectra were recorded in a positive mode, with ion energy of 0.3 eV and scan range of 0–1000 Da.

### 2.8. FT-IR Analysis

The oven was preheated to 160 °C. Liquid soy adhesives with or without cross-linkers were put in the oven to a constant weight. The cured soy adhesives were ground into fine powder. 1 g KBr and 0.01 g soy adhesive samples were mixed well for the preparation of KBr pills. The FT-IR spectra were gotten on a Varian 1000 infrared spectrophotometer (Varian, Palo Alto, CA, USA).

### 2.9. ^13^C-NMR

The 400 μL liquid sample was directly mixed with 50 μL DMSO-d6 for ^13^C-NMR determination. The spectra were obtained on a Bruker AVANCE 600 NMR spectrometer (Bruker Corporation, Billerica, MA, USA) using 12 μs pulse width (90°). The relaxation delay was 6 s. To achieve a sufficient signal-to-noise ratio, inverse-gated proton decoupling method was applied. The spectra were taken at 150 MHz with 400–600 scans accumulated.

## 3. Results and Discussion

### 3.1. The DSC and DMA Analysis of Soy-Based Adhesive with HPF

**Figure 1:**
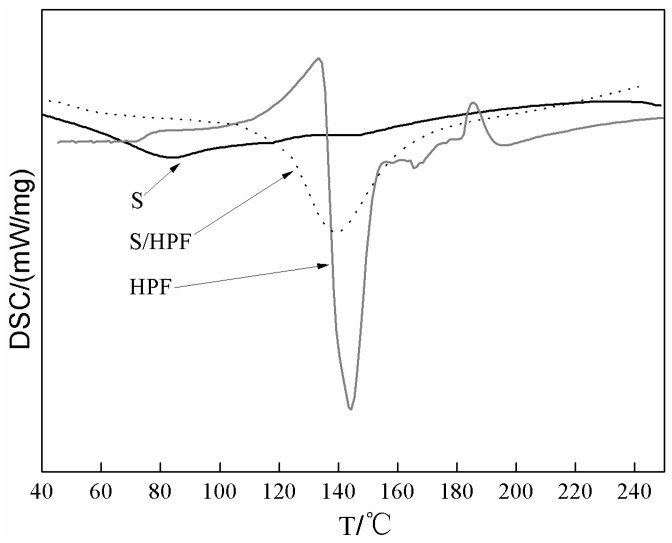
DSC results of soy-based adhesive (S), HPF and their mixture S/HPF.

Figure 1 was the DSC results of the soy-based adhesive, HPF and their mixture. The soy-based adhesive without cross-linker showed no obvious exothermal reaction. The soy-based adhesive with HPF showed an exothermal peak at a lower temperature, around 140 °C, than HPF itself at around 145 °C, which meant that the curing temperature of the former was lower than that of the latter. Since the addition amount of HPF was 12% of the solid soy flour while a similar quantity of heat was given off, the decrease of the curing temperature might be caused by the reaction between the soy-based adhesive and HPF.

**Figure 2:**
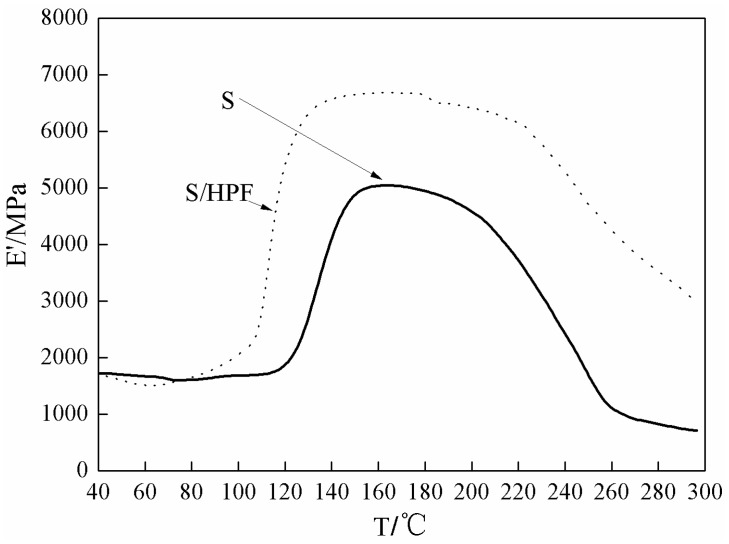
DMA results of soy-based adhesive and its mixture with HPF (S/HPF).

The DMA results of the soy-based adhesive with or without HPF cross-linker are given in Figure 2. Seen from Figure 2, the storage modulus (*E*’) for both samples increased abruptly at around 110 and 120 °C, respectively. Considering that the soy protein–based adhesive without HPF would not cure by itself as seen from Figure 1, the abrupt increase of the storage modulus for the soy adhesive sample without HPF might be caused by the drying of the adhesive. The lower temperature of the soy-based adhesive with HPF with the abrupt increase of *E*’ agreed well with the results of DSC and could be an indicator of the reaction between HPF and the soy-based adhesive. The curing reaction might be responsible for the better mechanical performance and heat resistance of the soy-based adhesive with HPF than that without HPF as well, which was indicated by its higher *E*’ and higher decomposition temperature.

### 3.2. The ESI-MS Analysis between HPF and AG

**Figure 3:**
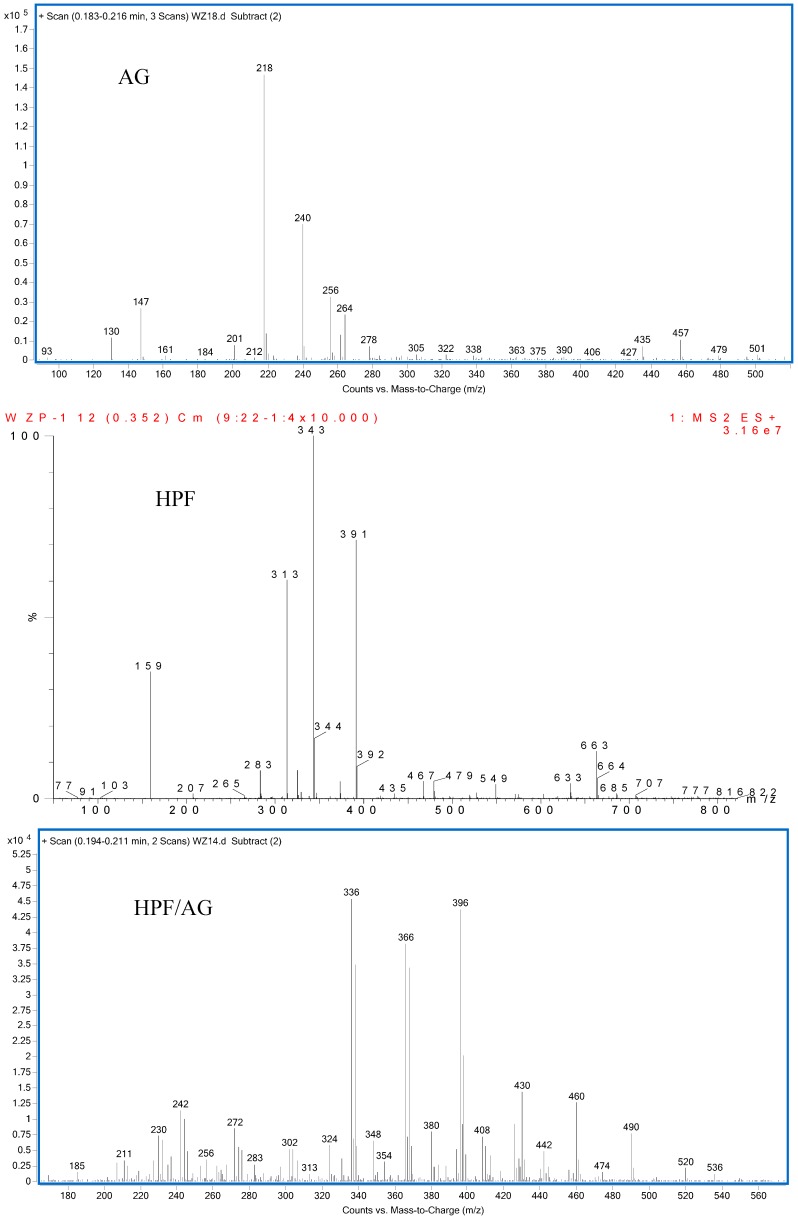
ESI-MS spectra of *N*-(2)-l-alanyl-l-glutamine (AG), HPF and HPF/AG.

The ESI-MS spectra of AG, HPF and HPF/AG are given in Figure 3. According to the mechanism of ESI-MS, the ion peaks observed in the ESI-MS spectra might come from ions in three forms, namely M + H^+^, M + Na^+^ and M + K^+^. If there is a nitrogen atom in the chemical group M, the ion peak will be observed mainly as a form of M + H^+^. Also, if there is an oxygen atom, it will be in forms of M + Na^+^ and M + K^+^ [12]. All of the three ion forms for the AG sample, 218, 240 and 256 Da, were observed because of the nitrogen and oxygen atoms in the AG molecules, as seen in Table 1.

For HPF, only the ions in forms of M + Na^+^ were observed. Its main ion peaks appeared at 313, 343, and 391 Da, which could be assigned as the dimers with two phenol molecules. It meant that the HPF used in this paper was mainly composed of molecules with low molecular weight. Some mono-hydroxymethyl phenol from the addition reaction from phenol and formaldehyde a with molecular weight of 159 Da could be detected.

The reaction between HPF and AG was proved by the ion peaks with a molecular weight of 336, 366, 396 Da, and others with a higher molecular weight. The peaks of 336, 366 and 396 Da with an interval of 30 Da came from the addition reaction of one molecule of AG and one molecule of methylophenol. The number of hydroxymethyl groups determined the final detected molecular weight. It must be noted that the structure in Table 1 only showed one possible structure which gave some information on the units and their numbers in one molecule. The isomeric compounds might exist.

The reactions between dimers in HPF and AG could be also clearly detected, as seen from ion peaks at 430, 460, 490 and 520 Da.

### 3.3. The FT-IR Analysis between HPF and AG

**Figure 4:**
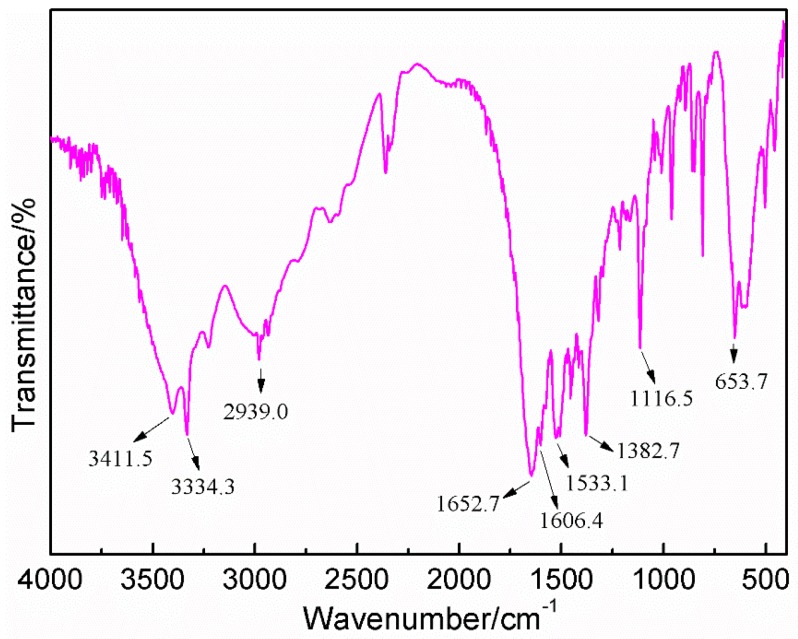
FT-IR spectrum of *N*-(2)-l-alanyl-l-glutamine.

The FT-IR spectrum of AG is given in Figure 4. The broad band observed at 3411.5 cm^−1^ was assigned to the stretching of the –NH_2_ groups. The stretching vibration band of –CH_2_–was observed at 2939.0 cm^−1^. The absorption at 1652.7 and 1606.4 cm^−1^ came from the stretching vibration of C=O from the –NH–C=O and O=C–NH_2_ groups, respectively. The C–N stretching from the –CO–NH–C group was observed at 1533.1 cm^−1^. The absorption at 1382.7 cm^−1^ was assigned as the C–O stretching vibration from the –COOH group. The absorption at 653.7 cm^−1^ came from N–H from the O=C–NH_2_ group [13].

**Figure 5:**
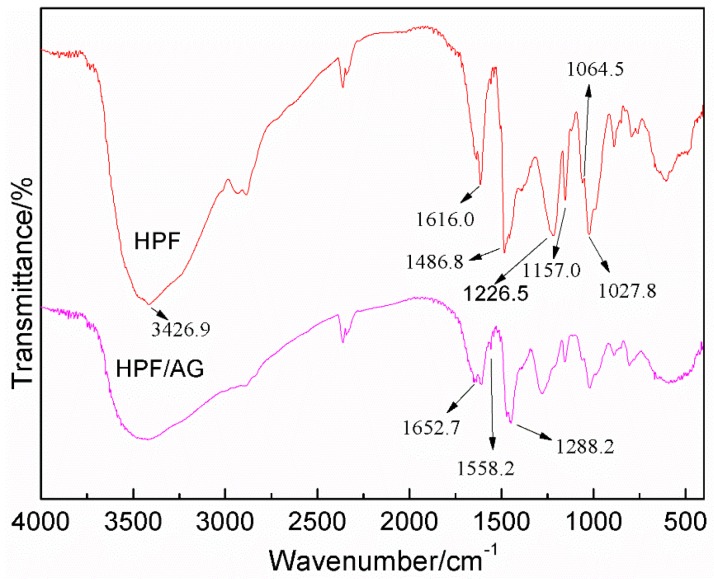
FT-IR spectra of samples HPF and HPF/AG.

The FT-IR results of the HPF and HPF/AG samples are given in Figure 5. Seen from Figure 5, the strong and broad peak at 3426.9 cm^−1^ was assigned to the stretching vibration of the –OH bond of the phenolic hydroxy group or the hydroxymethyl group. The C=C bond from the phenolic ring could be observed at 1616.0 and 1486.8 cm^−1^. The absorption at 1226.5 cm^−1^ could be assigned to the stretching vibration of the C–O from phenol. The absorption at 1157.0 cm^−1^ was the C–C stretching from the bond structure between the aromatic ring and hydroxymethyl group. The absorption at 1064.5 and 1027.8 cm^−1^ came from the C–O bond from the C–O–C of the phenolic resin and hydroxymethyl groups, respectively [14,15].

In the FT-IR of sample HPF/AG, the structure of C=O at 1652.7 cm^−1^ and C–N at 1558.2 cm^−1^ from *N*-(2)-l-alanyl-l-glutamine could be observed. The absorption peak from the C–O stretching vibration of the phenolic ring structure shifted from 1226.5 to 1288.23 cm^−1^. The shift was caused by the reaction between HPF and AG. Once the hydroxymethyl groups reacted with the reactive groups of dipeptides –NH_2_ or even –COOH, the cloud density of the phenolic ring would increase because of the conjugative effects between nitrogen or oxygen and the ring. Then the force constant of the C–O bond would increase and its vibration frequency would get lower.

### 3.4. The ^13^C-NMR Analysis between HPF and AG

**Figure 6:**
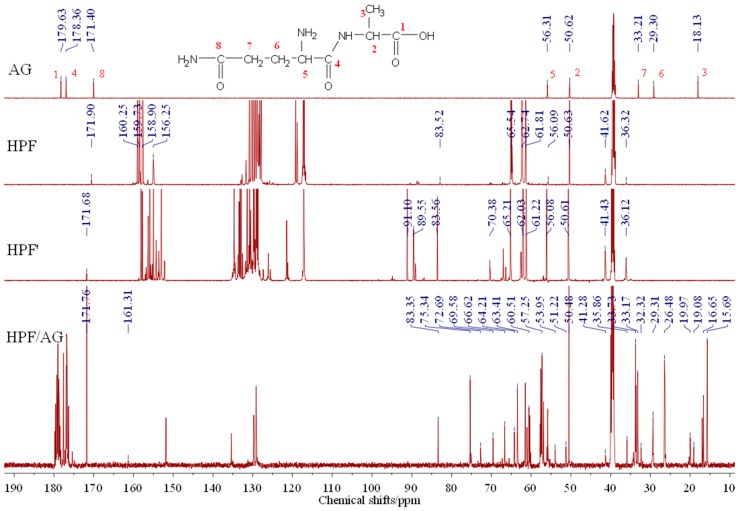
^13^C-NMR spectra of sample AG, HPF, HPF’ and HPF/AG.

The ^13^C-NMR results of AG, HPF and HPF/AG are given in Figure 6. The clarity of the ^13^C-NMR results of the AG sample reflected the purity of the sample used in this paper. All the carbons and their assignments in the AG sample are labeled in Figure 6.

Seen from the ^13^C-NMR results of the HPF sample, the chemical shifts at 61.82 and 62.74 ppm were assigned to *o*-CH_2_OH and that at 65.54 ppm was assigned to *p*-CH_2_OH. The shift at 115–135 ppm came from the carbon of the phenolic ring. The shift at 156.25–160.25 ppm was from the carbon of Ph–OH. The low polymerization degree of HPF could be seen by the low intensity of the methylene groups. Only two kinds of methylene groups were observed, specifically *p*-CH_2_-*p*′ at 41.62 ppm and *o*-CH_2_-*p* 36.32 ppm.

Seen from the ^13^C-NMR results of the HPF’ sample and the HPF sample, they had a very similar chemical structure. Both the –CH_2_OH and –CH_2_– groups from the addition and condensation reactions, respectively, could be seen. The new peak at 70.38 ppm could be assigned as the Ph–CH_2_–O–CH_2_–Ph groups, which were formed by the self-condensation of HPF under a higher temperature. The peaks from formaldehyde and its derivatives, seen at 83.56, 89.55 and 91.10 ppm, became more obvious in the HPF’ than in the HPF sample.

The main difference between the ^13^C–NMR results of HPF’ and HPF/AG came from the new appearance of peaks at 72.69 and 75.34 ppm. Both of them could be assigned as the methylene ether groups –CH_2_–O–CH_2_–. In fact, for a phenol-formaldehyde, seen from the ^13^C–NMR results of HPF’, the amount of the ether groups should normally not be as great as what was shown in the ^13^C–NMR results of HPF/AG. Especially, the appearance of the peak at 75.34 ppm could be direct proof of the reaction between HPF and AG, which could be assigned as branched ether groups [12]. In the system of the HPF sample being heated for 30 min, there would be no branched ether groups in existence. Considering the structure of HPF and AG, the ether groups at 75.34 ppm could be assigned as =N–CH_2_–O–CH_2_–N=, which came from the reaction between the methylol groups of HPF and the amide groups of AG. Besides ether groups, methylene groups –CH_2_– might be another structure occurring from the condensation reaction between HPF and AG. However, because of the overlapping of the peaks from 40 to 50 ppm from the -CH_2_- groups [16,17,18], the reaction between HPF and AG could not be determined by these groups. In the HPF/AG sample, the peak at 171.76 ppm coming from carbon 8 showed no obvious shift, which might be an indication of the lesser reaction or even lack of reaction of the aliphatic amino groups connected with carbon 8 of HPF under the experimental conditions introduced in this paper. Although the peak at 29.31 ppm coming from carbon 6 also showed no obvious shift, the peaks around the chemical shift of carbons 4, 5 and 7 became more complex. The reaction of the amino groups at carbon 5 was difficult to judge.

The carboxyl group –COOH is another possible reactive group in the AG sample. The shift from 179.63 to 179.21 ppm being assigned to carbon 1 of the AG sample seemed to be proof of the reaction between –COOH and HPF. However, on one hand, the almost unchanged chemical shift from carbon 2 appearing at around 50.6 ppm denied this speculation. On the other hand, the reaction between –COOH and HPF could not happen or would be neglected under a temperature as low as 80 °C.

## 4. Conclusions

The performance of soy-based adhesive with HPF and the reaction between HPF and the common dipeptide AG were studied in this paper. The DSC and DMA results indicated the reaction between the HPF and soy-based adhesive. The soy-based adhesive with HPF cured at a lower temperature than the adhesive without HPF. The former showed better mechanical performance and heat resistance than the latter. The reaction between HPF and AG was proved. Because of the existence of branched ether groups in the ^13^C–NMR results of HPF/AG, the reaction between HPF and AG might mainly happen between the hydroxymethyl groups and amino groups under a basic condition.

## Figures and Tables

**Table 1:** The main ion peaks of ESI-MS and their assignments.

Experimental (Da)				Chemical Species
Samples	M + H^+^	M + Na^+^	M + K^+^	
AG	218	240	256	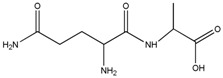
HPF	–	159	–	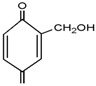
	–	283	–	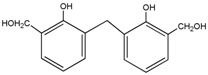
	–	313	–	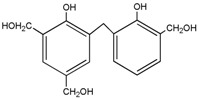
	–	343	–	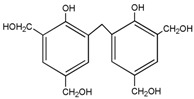
	–	391	–	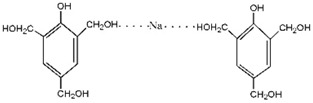
HPF/AG	336	–	–	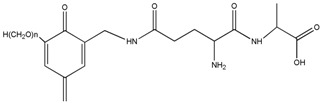 or 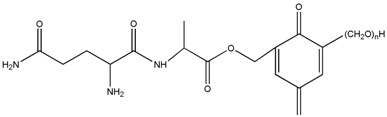
	366	–	–	
	396	–	–	
	430	–	–	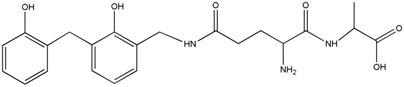
	460	–	–	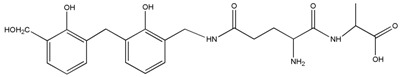
	490	–	–	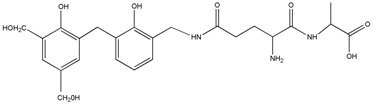
	520	–	–